# A Desecration of Florence Nightingale's Statue

**Published:** 1916-05-20

**Authors:** 


### A DESECRATION OF FLORENCE NIGHTINGALE'S STATUE.

We mentioned last week that the statue of Florence Nightingale in Waterloo Place had been removed from its base immediately before the anniversary of her birthday. The men doing the work said it would také a fortnight, but Mr. G. Q. Roberts, secretary to the Memorial Committee, approached the head of the firm of contractors, and he came up from Birmingham on Thursday; his men worked all Thursday night and succeeded in replacing the statue on Friday morning, though surrounded with a wooden structure, whilst its base was boarded in. Unfortunately, by some influence devoid of discretion and proper respect for Florence Nightingale's memory, the statue was allowed to be defaced and burlesqued, a fact demonstrated with cruel accuracy in the *Daily Graphic* illustration of the 13th instant. But then the "Lamp" Day as illustrated in the same paper reveals how largely movements of this kind may be used for the purpose of personal advertisement.

The protection of the sacred memory of the illustrious dead is a duty which imposes this protest upon us. It enforces the insult to the nursing profession which this desecration of the Nightingale statue in fact was. We are surprised to find the *Daily Telegraph* recording, if we read it aright, that this desecration was committed under the personal direction of Lady Cowdray, who divided her time on Lamp Day between the statue and her house in Carlton House Terrace. We hope that some amends will be made and an apology tendered. In any case it is essential that steps should be taken, through the First Commissioner of Works, to protect the statue of Florence Nightingale from any further desecration of the kind, on the occasion of her birthday or otherwise.

Again, it is utterly wrong that Florence Nightingale's name should be utilised by politicians and promiscuous Associations for the promotion of their propaganda and the raising of funds.

